# Enhanced Therapeutic Efficacy in Cancer Patients by Short-term Fasting: The Autophagy Connection

**DOI:** 10.3389/fonc.2016.00242

**Published:** 2016-11-14

**Authors:** Gustav van Niekerk, Suzèl M. Hattingh, Anna-Mart Engelbrecht

**Affiliations:** ^1^Department of Physiological Sciences, Stellenbosch University, Stellenbosch, South Africa; ^2^Department of Biomedical Sciences, Division of Medical Physiology, Faculty of Medicine and Health Sciences, Stellenbosch University, Tygerberg, South Africa

**Keywords:** autophagy, chemotherapy, fasting, immunogenic cell death, oncolytic virus

## Abstract

Preclinical studies suggest that fasting prior to chemotherapy may be an effective strategy to protect patients against the adverse effects of chemo-toxicity. Fasting may also sensitize cancer cells to chemotherapy. It is further suggested that fasting may similarly augment the efficacy of oncolytic viral therapy. The primary mechanism mediating these beneficial effects is thought to relate to the fact that fasting results in a decrease of circulating growth factors. In turn, such fasting cues would prompt normal cells to redirect energy toward cell maintenance and repair processes, rather than growth and proliferation. However, fasting is also known to upregulate autophagy, an evolutionarily conserved catabolic process that is upregulated in response to various cell stressors. Here, we review a number of mechanisms by which fasting-induced autophagy may have an impact on both chemo-tolerance and chemo-sensitization. First, fasting may exert a protective effect by mobilizing autophagic components prior to chemo-induction. In turn, the autophagic apparatus can be repurposed for removing cellular components damaged by chemotherapy. Autophagy also plays a key role in epitope expression as well as in modulating inflammation. Chemo-sensitization resulting from fasting may in fact be an effect of enhanced immune surveillance as a result of better autophagy-dependent epitope processing. Finally, autophagy is involved in host defense against viruses, and aspects of the autophagic process are also often targets for viral subversion. Consequently, altering autophagic flux by fasting may alter viral infectivity. These observations suggest that fasting-induced autophagy may have an impact on therapeutic efficacy in various oncological contexts.

## Introduction

A major hurdle in the fight against cancer is the narrow therapeutic window associated with cytotoxic chemotherapeutic agents. Strategies aimed at decreasing the toxic effect associated with chemotherapeutic interventions face the risk of similarly protecting cancer cells, thus abrogating the therapeutic effect of antineoplastic therapies. In this regard, short-term fasting has emerged as a potential intervention that may alleviate various side-effects associated with chemotherapy, without attenuating the antineoplastic efficacy of the chemotherapeutic agent. Initial studies have demonstrated that mice fasted for 48–60 h were protected against a lethal dose of etoposide, a chemotherapeutic with a notoriously narrow therapeutic window (1). This result was observed in three different mice strains and did not attenuate the therapeutic efficacy of etoposide against cancer cells (1). In a subsequent study (2), fasting conditions were shown to sensitize various cancers to a range of chemotherapeutics and even attenuate the growth of certain cancers *in vitro*. A clinical trial testing the feasibility of incorporating short-term fasting (72-h fasting around the time when the therapeutic agent is administered) into platinum-based chemotherapy (3) found fasting to be well tolerated. Moreover, results also indicated that fasting did not protect cancer cells. Similar encouraging results were obtained in another trial in HER2-negative breast cancer patients who fasted 24 h before and another 24 h after receiving chemotherapy (4). Although these were small pilot studies, initial results are promising.

Fasting is known to extend the life span of various organisms by acting on the same evolutionarily conserved nutrient-sensing pathways (5, 6). It is currently believed that the same pathways are involved in mediating the differential stress response to fasting between normal and cancerous cells. The underlying rationale is that, during the time of nutrient deprivation, an organism would flux resources away from growth and development and invest in maintenance and protective mechanisms. Consequently, a decrease in growth factors, such as IGF-I (1, 7), causes normal cells to downregulate proliferation and upregulate cell protective mechanisms. Thus, fasting induces the same protective mechanisms against a number of adverse effects of chemotherapeutic agents. However, recent findings now also implicate additional mechanisms. In this article, we review emerging evidence supporting a key role played by macroautophagy (hereafter referred to as autophagy) in not only attenuating the toxic effects of chemotherapy but also in enhancing the efficacy of antineoplastic therapies. In particular, we highlight the ability of fasting to enhance immune surveillance, suggesting that short-term fasting may provide best results in the context of chemotherapeutic agents that induce immunogenic cell death (ICD).

## Fasting-Induced Autophagy: Enhancing Cell Survival

Autophagy is an evolutionarily conserved system by which cells degrade long-lived cellular components. During bouts of fasting, autophagy is upregulated in order to “digest” non-critical cellular components into substrate to be used for energy production as well as synthesis and repair of critical housekeeping proteins. However, autophagy is also upregulated in response to various stressors (8). For example, autophagy promotes cell survival in response to heavy metals (9) and hypoxia (10) and is also protective in systemic insults, such as sepsis (11).

Similarly, an upregulation of autophagy prior to the induction of a toxic chemotherapeutic agent may be protective. As an example, chemotherapy directly, or *via* an increase in oxidative stress, causes damage to organelles, such as the mitochondria (12). Here, autophagy plays a critical role in quality control of mitochondrial networks, targeting defective mitochondria for degradation (13). Similarly, in a sterile-injury model (third-degree burn wounds in rabbits), mortality is dramatically increased when autophagy is inhibited (14). In particular, the accumulation of mitochondria with low membrane potential has been implicated as the causative agent in the pathology induced by autophagic insufficiency. Another key function of autophagy is to provide an alternative route for removing misfolded proteins when the proteasomal pathway is saturated. Indeed, endoplasmic reticulum (ER) stress, caused by an accumulation of misfolded proteins, also activates autophagy (15). Taken together, these observations suggest that a systemic upregulation of autophagy before chemotherapy is administered could be beneficial. Since fasting is a well-established mechanism for inducing autophagy, it seems likely that fasting-induced autophagy may play a critical role in limiting chemo-toxicity.

Fasting-induced upregulation of the autophagic process may render cells prepared to deal with a systemic challenge faced during chemotherapeutic induction. But importantly, this also raises the question whether fasting-induced autophagy may not similarly be protective to cancer cells. Indeed, the observation that inhibition of autophagy sensitizes cancer cells to therapeutic interventions demonstrates that autophagy also has a key pro-survival function in numerous cancers (16–18). However, cancer cells often demonstrate a comparatively lower capacity to induce autophagy, since the development of cancer is often associated with a defect in autophagic capacity. As an example, oncogenes that are often upregulated in cancer (e.g., Akt, PI3K, and Bcl-2 proteins) inhibit autophagy, whereas tumor suppressor genes often mutated or lost (e.g., DAPK1 and PTEN) are usually involved in the upregulation of autophagy (16, 17). This would suggest that, at least in some cancers, the differential stress response may in fact manifest from the inability of cancer cells to sustain autophagic processes as effectively as normal cells. Also, many growth factors potently inhibit autophagy by promoting the utilization of exogenous nutrients (19). The decrease in circulating growth factors may thus result in an upregulation of autophagy in normal cells, but not cancer cells that have developed insensitivity to external growth cues. Thus, there is reason to suspect that host cells, which are more responsive to fasting cues, may upregulate autophagy to a greater extent than cancer cells.

## Autophagy and Immunological Cell Death

Accumulating evidence indicates that the antineoplastic effect of various chemotherapeutic agents is not only dependent on their cytotoxic effects but also that such agents exert their therapeutic effect through their ability to reinstate immune surveillance after inducing ICD in cancer cells (20). Mechanistically, certain chemotherapeutics (e.g., mitoxantrone and anthracyclines such as doxorubicin) that induce ER stress enhance the expression of calreticulin on the cell membrane, which in turn promotes the engulfment of apoptotic bodies by antigen-presenting cells (21). Another hallmark of ICD is the release of danger-associated molecules (DAPMPs), such as ATP, various heat shock proteins, and signaling molecules (e.g., cytokines such as IL-1α) (22). In turn, the pro-inflammatory state induced by these DAMPs and inflammatory mediators promotes the maturation of dendritic cells and subsequent activation of CD4+ and CD8+ T-cells (22).

Interestingly, autophagy has also been shown to enhance the immunogenicity of dying tumor cells by secretion HMGB1 (23). Similarly, autophagy has been shown to play a critical role in mediating ICD through the release of ATP prior to apoptosis (24). This suggests that the recruitment of the autophagic machinery (e.g., by fasting) prior to administering chemotherapeutic agents known to induce ICD may enhance the therapeutic efficacy of these agents. Strikingly, recent findings show that this may indeed be the case. Fasting (48 h) or administering agents known to upregulate autophagy (hydroxycitrate) augments the antineoplastic properties of mitoxantrone and oxaliplatin, an effect which was not observed if cancer cells were implanted in immune-compromised mice, or if the critical autophagic gene *Atg5* was silenced (25). In particular, the role of autophagy-mediated ATP release is demonstrated by two observations. First, in autophagy-deficient cells, ATP release is attenuated. Second, cancer cells overexpressing CD39, an ecto-ATPase that rapidly converts ATP to AMP, demonstrated a marked decrease in chemotherapeutic efficacy, which was reversed by intra-tumor administration of ATPase inhibitors. The observation that fasting-induced autophagy promotes ICD suggests novel mechanisms through which fasting may enhance chemotherapeutic efficacy. Since autophagy is involved in the secretion of other ICD agents (26), future studies may investigate whether fasting may similarly “prime” cells for autophagy-mediated release of ICD elicitors.

Furthermore, autophagy also plays a critical role in epitope expression. The prototypical view where MHC II epitopes are derived from exogenous (i.e., phagocytosed) sources and MHC I from endogenous sources has been supplemented by findings indicating that phagocytosed particles could be expressed on MHC I (27), and conversely, that endogenous epitopes can be expressed by MHC II (28). While autophagy has a more established role in the expression of epitopes by MHC II (29), recent evidence has implicated autophagy in the expression of epitopes by MHC I (30). Macroautophagy provides a constant source of cytosolic proteins that fuse with MHC II loading compartments (29), suggesting that fasting-induced autophagy may increase the presentation of cytosolic epitopes. Supporting this view, autophagy, induced *in vitro* by starvation, enhances the expression of intracellular proteins on MHC II (31). Short-term fasting has also been shown to enhance the mucosal-derived B lymphocyte response to an orally administered influenza vaccine (32). Similarly, administration of the mTOR-inhibitor rapamycin enhances vaccine efficacy by promoting cross-strain protection (33). Similar to the effects of autophagy induction in enhancing vaccine efficacy, upregulating autophagy prior to chemotherapy may enhance immunization against tumor antigens.

In fact, there is evidence that autophagy may play an important role in epitope presentation within an oncological context. As an example, autophagy, induced by either starvation or pharmacologically in antigen-donating cancer cells, enhances the cross-presentation of these antigens by dendritic cells (34). Also, recent findings suggest that a fusion construct consisting of a cancer antigen (NY-ESO-1) with proteins targeting the cancer antigen for autophagic degradation could assist in epitope expression by MHC II and enhancement of CD4+ T cell-mediated antitumor responses (35). These observations suggest that the role of autophagy in managing various infections and promoting vaccine efficacy are likely also important in reinstating anticancer immune response. This then also suggests that fasting may mediate therapeutic effects by enhancing epitope expression resulting from an upregulation of autophagic processes.

Initial findings from preclinical studies suggests that fasting my sensitize cancer cells to chemotherapeutic interventions (2). From the preceding discussion, it appears that an upregulation of autophagy may mediate such a “chemo-sensitizing” response by enhancing epitope presentation and augmenting the reinstatement of immune surveillance. However, autophagy may also attenuate an inflammatory response and contribute toward cancer cells’ ability to evade immune effectors. As an example, though autophagy has been implicated in the unconventional secretion of IL-1β (36), a well-known pro-inflammatory cytokine, autophagy may also inhibit the expression of IL-1β production by targeting the activated inflammasome for autophagic degradation (37). In addition, autophagy may also promote immune evasion by cancer cells. As an example, inhibition of autophagy in *HRAS*-transformed mouse kidney endothelium results in an altered proteome with a more pro-inflammatory profile, “priming” cells for an innate immune response (38). Similarly, inhibition of autophagy has also been shown to promote ICD in at least some tumors (39). Autophagy has also been implicated as a mechanism by which cancer cells avoid NK-mediated lysis by targeting granzyme B released for autophagic degradation (40). These observations demonstrate that autophagy may also play a role in mediating immune evasion by transformed cells.

## Oncolytic Viruses

Recently, evidence has emerged that fasting may also enhance the efficacy of oncolytic viruses (OVs) (41). In normal cells, fasting increases the phosphorylation status of translation initiation factor eIF2α, resulting in a depression of protein synthesis; in contrast, cancer cells do not exhibit the same level of eIF2α phosphorylation. Consequently, viral growth is inhibited in fasting-responsive normal cells as they downregulate the translational machinery required for viral replication, while cancer cells remain insensitive (41). This may explain the sensitivity of cancer cells to the herpes simplex virus (HSV) compared to host cells when administered in a fasted state (41). It is likely that fasting may also induce similar results in other OVs. Since viruses are dependent on host replication machinery, fasting would suppress viral replication by downregulating cellular replication and translational activities. In contrast, cancer cells that are less responsive to fasting cues would maintain higher metabolic activity and thus be more susceptible to viral infections.

However, fasting-induced autophagy may also have an impact on oncolytic viral efficacy, since autophagy is also implicated in the host’s immune response toward viral infections (26, 42). As an example, upregulating autophagy by means of fasting results in a dramatic suppression of viral infections in a variety of cell types (43). This would suggest that fasting may preferentially sensitize autophagy-deficient cancer cells to HSV, as normal cells would be more resistant by virtue of the elevated levels of autophagy in cells responding to a fasted state. Of note, an upregulation of autophagy may not be beneficial to all types of viruses since certain viral agents also subvert autophagic processes for replication and the non-lytic release of viral particles (44). As an example, the hepatitis C virus is known to upregulate autophagy in hepatocytes, where the inhibition of autophagy in fact hampers viral replication as cells induce apoptotic cell death (45). Indeed, the key role played by autophagy in cell-autonomous defense against viral infections has also generated interest as pathways that may be targeted for enhancing OV efficacy (46). An upregulation of autophagy through fasting may thus either increase or decrease viral infectivity, since autophagy has different roles in the replication strategies of OVs.

Furthermore, viral subversion of autophagic processes is known to depend on the tissue type being infected (44). This would suggest that the tissue of origin in cancer may also have an impact on the outcome of fasting-induced autophagy in OV therapy. Intriguingly, recent evidence suggests that the evolution of drug resistance may also alter the role of autophagy in viral replication (47). Mice inoculated with A549 cancer cells that were either resistant to cisplatin (A549/DDP) or paclitaxel (A549/PTX) demonstrated contrasting tumor growth rates in response to autophagy modulation: whereas the autophagy inhibitor chloroquine enhanced viral-induced cell death of A549/DDP cancer cells, the inhibition of autophagy by rapamycin rendered A549/PTX cells more susceptible to viral lysis. Thus, both tissue origin and the development of drug resistance are likely to have an impact on clinical trials where the role of fasting in OV therapy is evaluated.

Autophagy as a catabolic process plays a key immunological role in enhancing cell-autonomous defenses, and, as such, pathogens such as viruses have evolved various strategies both to inhibit and to subvert this process (44, 48). Consequently, it is likely that upregulation of autophagy may not be effective for all OVs or even counterproductive. However, the role of autophagy in modulating viral infectivity also suggests that the genetic manipulation of OVs to render them more “autophagy-sensitive” might be an attractive strategy for treating autophagy-deficient cancers.

**Figure 1:**
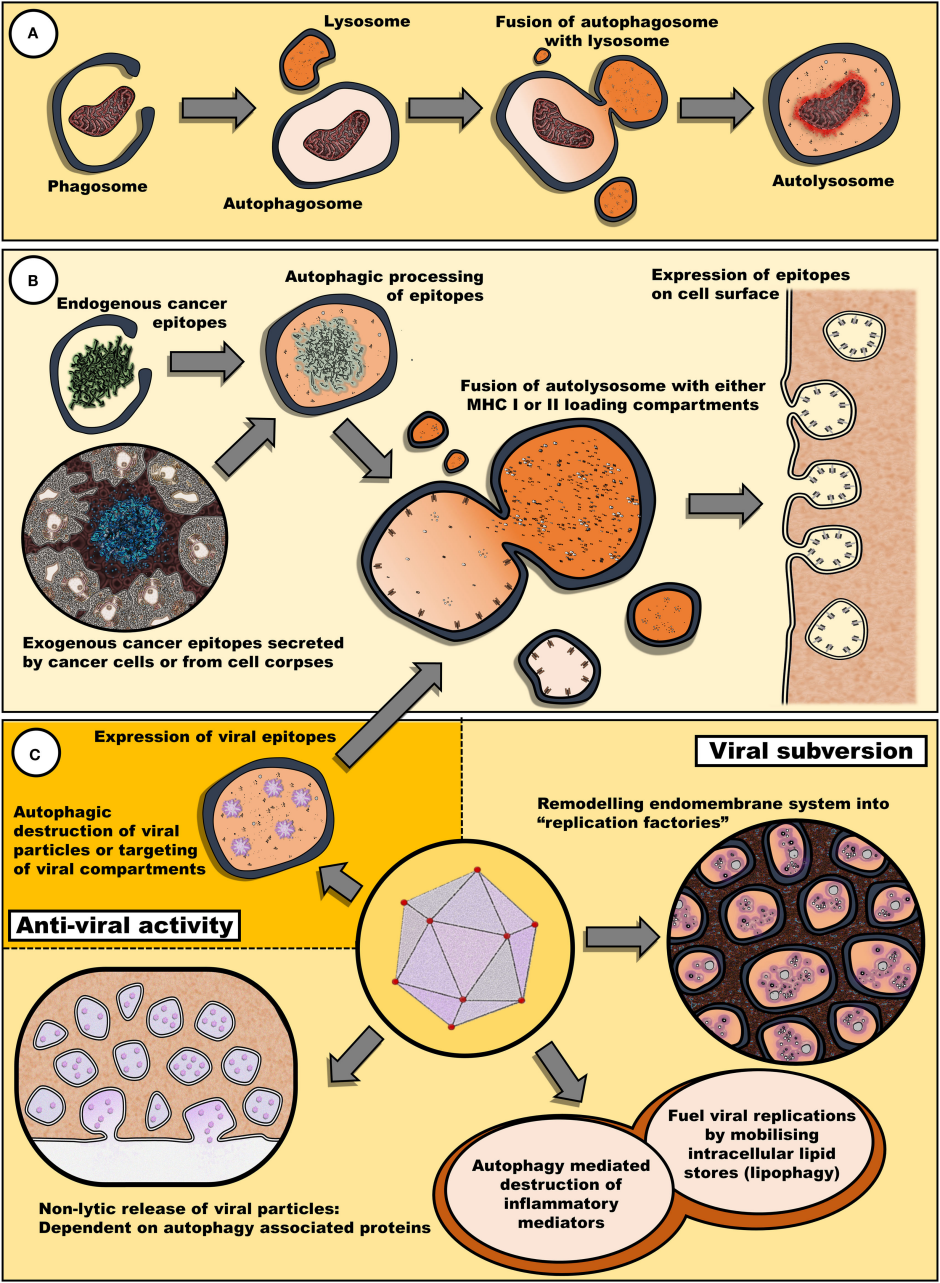
**Autophagy may remove cellular components damaged by antineoplastic therapies and also promote the reinstatement of immune surveillance against cancer cells**. **(A)** Autophagy plays a key role in the clearance of damaged cellular components and organelles. Removal of protein aggregates or damaged mitochondria may promote cell survival. **(B)** Autophagy is also involved in epitope expression. Exogenous antigens derived from cell bodies or secreted by cancer cells can be processed into epitopes to be loaded in MHC I and II loading compartments. Similarly, endogenous epitopes processed by autophagy can be expressed by cancer cells, enhancing their immunogenic profile. **(C)** Fasting may also have an impact on the efficacy of oncolytic viral therapy. Many viruses target diverse steps within the autophagic process for either viral subversion or inhibition of host antiviral response [reviewed by Chiramel et al. (42)]. Thus, autophagy may either promote or impede viral infectivity. Not illustrated, autophagy has also been implicated in the release of inflammatory mediators, such as IL-1β, ATP, and HMGB1, *via* secretory autophagy (26).

Therefore, it appears that fasting may have an impact on both viral and chemotherapeutic interventions *via* diverse mechanisms involving both an upregulation of autophagy and a decrease in circulating growth factors (Figure 1).

## Unresolved Issues

Observations in mice suggest that fasting may represent a cost-effective intervention that may greatly increase the therapeutic window. However, it is not obvious how fasting protocols for mice would translate to humans. Mice have a large surface-to-body ratio with a metabolic rate vastly higher than that of humans (49); therefore, the effect of 48 h of fasting in mice would have far more pronounced effects in a rodent model compared to humans. As an example, the rate of hepatic gluconeogenesis in mice is about 11.4 times higher than in humans (49), suggesting that even 48-h fasting for mice may in fact greatly upregulate catabolic processes in order to maintain glucose levels. This pronounced effect of fasting in mice is demonstrated by the observation that 72 h of fasting induces a ~20% reduction in body weight (7). This raises the question whether more extreme fasting protocols need to be implemented in patients to replicate more accurately the metabolic consequences seen in mice. Prolonged fasting protocols that are necessary to induce a similar catabolic state in humans may not be feasible in many cancer patients who are already undernourished and lack the reserve capacity to tolerate prolonged bouts of fasting. These considerations thus highlight the need to understand the mechanisms by which fasting may benefit patients. As an example, the involvement of autophagy as a key mediator behind the effect of fasting would predict that prolonged fasting may not be necessary, as autophagy is rapidly upregulated even after short-term fasting (50). Also, the pharmacological activation of key pathways involved in the fasting response is an attractive alternative to direct fasting.

Additional studies are needed to identify the context in which fasting may be beneficial. As mentioned, there are contrasting findings on the role of autophagy in ICD. Furthermore, it has been suggested that other interventions, such as radiotherapy, may also benefit from fasting regimes (51). In this regard, radiotherapy has also been shown to induce ICD in a number of tumors (52). Since autophagy can either inhibit or promote ICD, it is likely that fasting-induced autophagy may also alter the therapeutic outcome of radio therapy.

Fasting may also invoke mechanisms beyond modulating nutrient and growth factor levels. As an example, postprandial reabsorption of bile acids acts as singling molecules that could alter the effects of chemotherapy. Bile acids alter a range of immune and metabolic activities *via* farnesoid X receptor (FXR) (53). Even in a fasted state, the pharmacological activation of FXR suppresses autophagy by attenuating the transcription of autophagic genes (54). In addition, FXR also has an anti-inflammatory function (55). As an example, FXR activation leads to the downregulation of monocyte chemoattractant protein-1 in macrophages (56). It remains to be established whether FXR signaling also modulates other peripheral immune effects. However, this does demonstrate that bile released in response to feeding may inhibit autophagy and also cause anti-inflammatory activity that may antagonize the mobilization of a robust immune response to cancer cells.

## Conclusion

In this article, we have highlighted the potential protective role of fasting-induced autophagy as a mechanism by which cells cope with the adverse effects of chemotherapy. In addition, we have pointed out that since autophagy is also involved in immune responses it is possible that an upregulation of autophagy may have an impact on immune function and, in particular, help or hinder the reinstatement of immune surveillance against transformed cells. Furthermore, fasting results in a range of physiological adaptations which may have an impact on the efficacy of chemotherapeutic agents. It is likely that the protective effects of fasting may be mediated by these processes operating in concert. A clear understanding of the mechanism invoked by fasting may lead to the development of pharmacological strategies that replicate these same processes. Such therapies may be of particular importance in patients who are already undernourished or where nutrient deprivation is contraindicated.

## Author Contributions

GN, SH, and A-ME wrote the manuscript. All the authors read and approved of the final version of the manuscript.

## Conflict of Interest Statement

The authors declare that the research was conducted in the absence of any commercial or financial relationships that could be construed as a potential conflict of interest.
